# Preventing feedback activation of glycolytic ATP production enhances metformin cytotoxicity in breast cancer cells when oxidative phosphorylation is inhibited

**DOI:** 10.1186/2049-3002-2-S1-P89

**Published:** 2014-05-28

**Authors:** Yongxian Zhuang, Daniel K Chan, W Keith Miskimins

**Affiliations:** 1 Cancer Biology Research Center, Sanford Research/USD, Sioux Falls, SD, USA

## Background

Metformin has been shown to inhibit cell proliferation and induce cell death in many cancer cell lines. However, some cell lines exhibit resistance to the cytotoxic effects of metformin in tissue culture medium with high glucose levels (25 mM). Recent studies suggest when glucose is lowered; metformin becomes increasingly cytotoxic to cancer cells. However, the precise mechanism for metformin’s cancer cytotoxicity remains unidentified.

## Materials and methods

*Cell death assay using Sytox Green Nucleic Acid Stain* Sytox Green nucleic acid stain (10 μM) was added directly to cells in 96 well plates and read to obtain dead cells. Total cell number was obtained after adding 0.4% Triton X-100.

*ATP assay* 10 μl lysate of equal numbers of cells was used following the manufacturer’s protocol for ATP assays from Invitrogen (ATP Determination Kit, A22066).

*Lactate Assay* Medium was tested for lactate concentration using L-Lactate Assay Kit (Eton Biosciences Inc.). Relative lactate level was obtained after normalized by total cell number.

*Measurement of Extra Cellular Acidification Rate (ECAR) and Oxygen Consumption Rate (OCR)* ECAR and OCR were measured using a Seahorse XF24 analyzer according to manufacturer’s instructions (Seahorse Bioscience). ECAR and OCR were plotted after normalizing to total cell number.

## Results

Metformin became more cytotoxic in all of the tested breast cancer cell lines when glucose concentration was titrated to low levels in tissue culture medium. Metformin enhanced cytotoxicity in low glucose medium was correlated with significant reduction of ATP that was not observed in high glucose containing medium. Supplementing low glucose medium with fructose or galactose failed to prevent the reduction of ATP or to rescue the cells from metformin induced cell death. This suggested that metformin treated breast cancer cells cannot efficiently use fructose or galactose to maintain ATP. Metformin is known to inhibit complex I and prevent ATP production. However, in high glucose containing medium metformin treated cells maintained ATP levels via feedback activation of glycolysis. This enhanced glycolysis was either diminished or blocked when metformin treated cells were switched to low glucose, high fructose or high galactose medium. These findings suggest that lowering glucose potentiates metformin induced cell death by reducing metformin stimulated glycolysis.

## Conclusions

Our data support a model in which metformin treatment of cancer cells in low glucose medium leads to cell death by decreasing ATP production via diminishing or preventing metformin stimulated glycolysis.

